# Revisiting bacterial cytolethal distending toxin structure and function

**DOI:** 10.3389/fcimb.2023.1289359

**Published:** 2023-11-14

**Authors:** Henry Chen, Claire J. Ang, Molly K. Crowder, William M. Brieher, Steven R. Blanke

**Affiliations:** ^1^ Department of Microbiology, School of Molecular and Cellular Biology, University of Illinois at Urbana-Champaign, Urbana, IL, United States; ^2^ Department of Cellular and Developmental Biology, University of Illinois at Urbana-Champaign, Urbana, IL, United States; ^3^ Department of Pathobiology, College of Veterinary Medicine, University of Illinois at Urbana-Champaign, Urbana, IL, United States; ^4^ Biomedical and Translational Sciences Department, Carle-Illinois College of Medicine, University of Illinois, Urbana, IL, United States

**Keywords:** AB toxin, cytolethal distending toxin, protein-protein interactions, *Campylobacter jejuni*, DNA damage, holotoxin structure

## Abstract

Cytolethal distending toxins (CDTs) are intracellular-acting bacterial genotoxins generated by a diverse group of mucocutaneous human pathogens. CDTs must successfully bind to the plasma membrane of host cells in order to exert their modulatory effects. Maximal toxin activity requires all three toxin subunits, CdtA, CdtB, and CdtC, which, based primarily on high-resolution structural data, are believed to preassemble into a tripartite complex necessary for toxin activity. However, biologically active toxin has not been experimentally demonstrated to require assembly of the three subunits into a heterotrimer. Here, we experimentally compared concentration-dependent subunit interactions and toxin cellular activity of the *Campylobacter jejuni* CDT (*Cj*-CDT). Co-immunoprecipitation and dialysis retention experiments provided evidence for the presence of heterotrimeric toxin complexes, but only at concentrations of *Cj-*CdtA, *Cj-*CdtB, and *Cj-*CdtC several logs higher than required for *Cj-*CDT-mediated arrest of the host cell cycle at the G_2_/M interface, which is triggered by the endonuclease activity associated with the catalytic *Cj-*CdtB subunit. Microscale thermophoresis confirmed that *Cj*-CDT subunit interactions occur with low affinity. Collectively, our data suggest that at the lowest concentrations of toxin sufficient for arrest of cell cycle progression, mixtures of *Cj-*CdtA, *Cj*-CdtB, and *Cj*-CdtC consist primarily of non-interacting, subunit monomers. The lack of congruence between toxin tripartite structure and cellular activity suggests that the widely accepted model that CDTs principally intoxicate host cells as preassembled heterotrimeric structures should be revisited.

## Introduction

The cytolethal distending toxins (CDTs) are a broadly distributed family of intracellular-acting genotoxins produced by mucocutaneous pathogens of the γ- and ε-Proteobacteria (Pickett and Whitehouse, 1999; Lai et al., 2021). CDTs have been isolated and functionally characterized from multiple pathogens, including *Aggregatibacter actinomycetemcomitans* (*Aa*), *Hemophilus ducreyi (Hd)*, *Escherichia coli (Ec)*, and *Campylobacter jejuni* (*Cj*) (Scott and Kaper, 1994; Cope et al., 1997; Whitehouse et al., 1998; Mayer et al., 1999). Within the extracellular environment, CDTs bind and are taken up into host cells, ultimately resulting in DNA damage, activation of the DNA damage response, and arrest of cell cycle progression (Guerra et al., 2011). Phosphatidylinositol-3,4,5-triphosphate phosphatase activity has also been associated with *Ec*-CDT, *Cj*-CDT, *Hd*-CDT, and *Aa*-CDT (Shenker et al., 1999; Gargi et al., 2013; Huang et al., 2021). Although increasing evidence implicates individual CDTs as important determinants of virulence (Fox et al., 2004; Jain et al., 2008; Pokkunuri et al., 2012), the structure-function relationships that underlie toxin interactions with host cells remain incompletely understood.

Most CDTs comprise three distinct subunits, CdtA, CdtB, and CdtC, which are encoded by contiguous genes within a single operon (Gargi et al., 2012). Analogous to most intracellular-acting bacterial protein exotoxins, CDTs are believed to possess classic “A-B” functional architecture, where the CdtA and CdtC subunits together are thought to constitute the “B component”, which facilitates the binding, uptake, and intracellular trafficking of the “catalytically active A component” (Blanke, 2006), CdtB, within host cells (McSweeney and Dreyfus, 2004; Damek-Poprawa et al., 2012; Eshraghi et al., 2014; Robb Huhn et al., 2021). High-resolution structural data indicate that, at high concentrations (>100 μM), the CdtA, CdtB, and CdtC subunits of *Hd*-CDT (Dragana Nesic and Stebbins, 2004) and *Aa*-CDT (Yamada et al., 2006) are assembled into heterotrimeric complexes, leading to a widely accepted model that these three subunits assemble into a functional, oligomeric holotoxin complex. Nonetheless, the importance of an assembled tripartite toxin complex for CDT cellular activity has not been definitively established.

Herein, we describe studies designed to evaluate the importance of the CDT tripartite structure for the cellular activity of the toxin from the human intestinal pathogen *Campylobacter jejuni* (*Cj*-CDT). Human epidemiological data coupled with animal infection studies support a role for *Cj*-CDT as an important determinant of pathogen colonization and virulence (Fox et al., 2004; Jain et al., 2008). However, the mechanisms by which *Cj*-CDT is assembled and secreted from *C. jejuni* resulting in functional toxin have not been reported. Also, the structure of functional *Cj*-CDT generated and released by the bacterium prior to intoxication of host cells has not been experimentally resolved. Strikingly, the isolation and purification of *Cj*-CDT from *C. jejuni* in culture has never been reported which makes it challenging to effectively carry out studies to delineate toxin structure-function relationships. Using three different experimental approaches, our studies here suggest that at concentrations at which *Cj-*CDT induces the arrest of cell cycle progression in mammalian cells, the three subunits (*Cj*-CdtA, *Cj*-CdtB, and *Cj*-CdtC) exist in solution as predominantly non-assembled monomers. These results suggest that the existing paradigm that *Cj*-CdtA, *Cj*-CdtB, and *Cj*-CdtC functionally interact with host cells as a preassembled tripartite toxin should be revisited.

## Experimental procedures/methods

### *Cj*-CDT expression and purification

Recombinant forms of *Cj*-*C*dtA, *Cj*-CdtB, *Cj*-CdtC were generated and purified as previously described (Eshraghi et al., 2010). Subunit purity was evaluated using sodium dodecyl sulfate polyacrylamide gel electrophoresis (SDS-PAGE) (Biorad, Hercules, CA) followed by Coomassie Brilliant Blue staining (Sigma, St. Louis, MO), and quantified using the Pierce BCA protein assay (Thermo, Rockford, IL).

### Removal of polyhistidine fusion peptides

His-tagged recombinant subunits were incubated at 21 ˚C with biotinylated thrombin (Novagen, Billerica, MA). After 20–24 h, biotinylated thrombin and cleaved polyhistidine peptides were removed using Pierce streptavidin agarose beads (ThermoFisher, Waltham, MA) and TALON Metal Affinity Resin (TaKaRa, Mountain View, CA), respectively. The beads and resin were removed using Spin-X centrifuge tube filters (pore size, 0.22 µm; Corning Costar, NY). *Cj*-CDT subunits free of polyhistidine fusion peptides, were quantified using the Pierce BCA assay. Polyhistidine removal was confirmed using SDS-PAGE and Coomassie Brilliant Blue staining.

### Mammalian cell culture

Human cancer colonic epithelial cells (HCT116, ATCC, Manassas, VA) were maintained in McCoy’s 5a Modified Medium (Corning, Manassas, VA) supplemented with 10% fetal bovine serum (Sigma, St. Louis, MO) and cultivated at 37°C and under 5% CO_2_ within a humidified environment.

### Cell cycle phase determination

*Cj*-CDT-dependent arrest of cell cycle progression at the G_2_/M interface was assessed using flow cytometry (FACSymphony A1, BD Biosciences, Franklin Lakes, NJ) as previously described (Eshraghi et al., 2010; Gargi et al., 2013).

### Dialysis retention assays

Purified *Cj*-CDT subunits were incubated together on ice or 37°C. After 1 h, the mixtures were dialyzed at 37°C against PBS pH 7.4 (1:1000 sample to buffer ratio), using Micro Float-A-Lyzer Dialysis Devices with a Molecular weight cutoff (MWCO) of 50 kDa (Spectrum Labs, CA). After 48 h, samples were harvested and evaluated for retention of *Cj*-CdtB using immuno-blot analyses.

### Immunoblotting

Following SDS-PAGE, resolved proteins were transferred to PVDF membranes (Millipore Sigma, Burlington, MA) using a wet/tank blotting system (Bio-Rad). Membranes were blocked with 5% bovine serum albumin (Sigma-Aldrich, St. Louis, MO) in TBS-T (0.1% Tween-20 in TBS pH 7.4, Fisher, Fair Lawn, NJ) and incubated with primary antibodies. Primary antibodies specific for each *Cj*-CDT subunit were generated commercially against peptide sequences unique to *Cj*-CdtA, *Cj*-CdtB, and *Cj*-CdtC (YenZme, San Francisco, CA) as follows: antibodies specifically targeting the *Cj*-CdtA-specific sequence 255-CPFTAKPLYRQGEVR-268, the *Cj*-CdtB-specific sequence 185-CDFNRDPSTITSTVDRELANR-204, and the *Cj*-CdtC-specific sequence 44-CFRDTSKDPIDQNWNIK-59. Membranes were then incubated with anti-rabbit IgG biotinylated antibodies (Cell Signaling, Danvers, MA), and subsequently with anti-biotin HRP-linked antibodies (Cell Signaling, Danvers, MA). Immunoblots were imaged using the ChemiDoc system (XRS+, Bio-Rad, Hercules, CA) following exposure to a 1:5 mixture of SuperSignal West Femto Maximum Sensitivity: Pico Plus Chemiluminescent Substrates (Thermo, Rockford, IL). Immunoblot densitometry analyses were performed using Image Lab software (Bio-Rad, Version 6.0).

### Microscale thermophoresis analysis

Polyhistidine-tagged *Cj*-CDT subunits, which had been labeled with NTA – Atto 647 N dye (NanoTemper, München, Germany), were incubated at 37°C and with non-polyhistidine-tagged, non-labeled, non-cognate subunits. After 1 h, samples were loaded into capillary tubes (Monolith NT.115 Series capillaries, NanoTemper, München, Germany) and placed into the microscale thermophoresis instrument (Monolith NT.115, software version 1.2.1, NanoTemper, München, Germany). Samples were allowed to equilibrate, in the instrument, for an additional 15 min at 37°C before collecting data. All readings were taken using the MO.Control program (version 1.6.1, NanoTemper, München, Germany) using red excitation (650 nm, 30-100% power), and medium MST power (40%). MST values were determined, at the 3 second temperature jump. Data were normalized to the fraction of complexed molecules (*FB*) as previously described (Zillner et al., 2012). MST values were fit to a log [*Cj*-CDT subunit] vs response equation to generate binding curves (GraphPad Prism version 8.1.2).

### *Cj-*CDT co-immunoprecipitation

Co-immunoprecipitation was conducted using antibodies bound to protein A magnetic beads (Dynabeads, Invitrogen, Waltham, MA). Purified *Cj*-CDT subunits were incubated on ice. After 1 h, the mixtures were further incubated at 4°C with the indicated antibodies. Immunoprecipitation (IP) antibodies were generated from sera obtained from rabbits immunized with full length recombinant *Cj*-CDT subunits (Immunological Resource Center, Univ. Illinois, Urbana, IL). After 24 h, the bound proteins were recovered by incubating beads in NuPAGE sample reducing agent plus LDS sample buffer (Invitrogen, Norway). Recovered *Cj*-CDT subunits were analyzed using SDS-PAGE followed by immunoblot analyses.

### Statistical analyses

Each experiment was performed at least three independent times. Error bars represent standard deviations. Statistical analyses were performed using GraphPad Prism 8.1.2. Dose response curves were fit to a log (agonist) vs response (three parameters) equation. R^2^ values indicate fit of the data to the regression model. Analysis of statistical differences was performed using one-way ANOVA followed by the Tukey’s *post-hoc* test. Statistical significance (*P* < 0.05) was determined at α = 0.05.

## Results

### *Cj*-CdtA, *Cj*-CdtB, and *Cj*-CdtC are required for maximal *Cj*-CDT cellular activity

**Figure 1:**
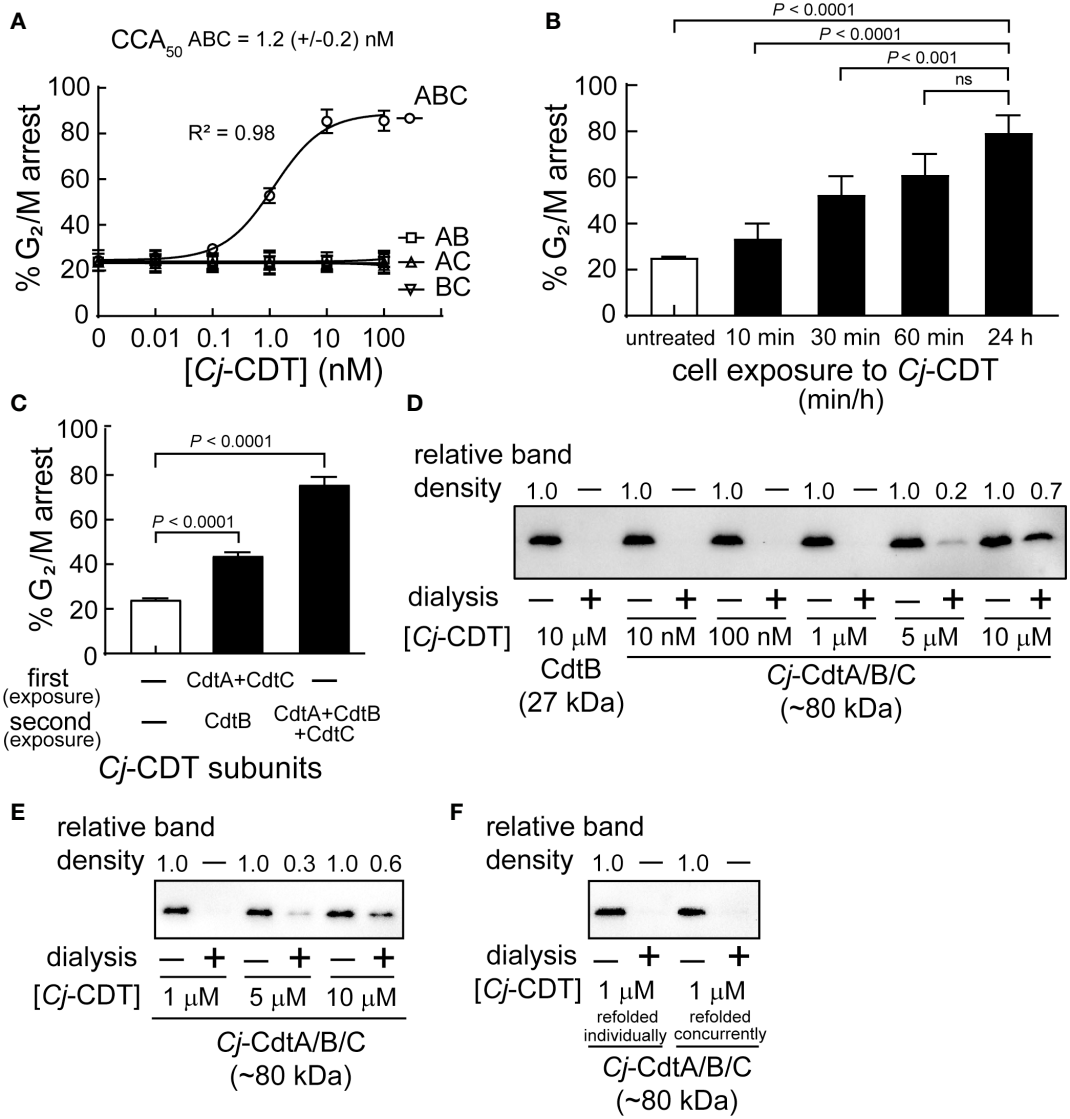
High molecular weight *Cj*-CDT tripartite structures are not captured during dialysis of biologically active mixtures of *Cj*-CDT subunits. **(A)** HCT116 cells were incubated in McCoy’s 5A medium + 10% FBS at 37°C and under 5% CO_2_ in the absence or presence of *Cj*-CDT (ABC), *Cj*-CdtA + *Cj*-CdtB (AB), *Cj*-CdtA + *Cj*-CdtC (AC), or *Cj*-CdtB + *Cj*-CdtC (BC) (10 pM – 100 nM). 24 h after the initial intoxication, cells were harvested and evaluated for cell cycle arrest progression. **(B)** HCT116 cells were incubated in the absence or presence of *Cj*-Cdt (ABC) (10 nM). After 10, 30, 60 min, or 24 h, cells were washed twice to remove unbound *Cj*-CDT, and further incubated at 37°C. 24 h after the initial intoxication, cells were harvested and evaluated for cell cycle arrest progression. **(C)** Pre-chilled HCT116 cells were incubated on ice in the absence or presence of *Cj*-CdtA and *Cj*-CdtC subunits (100 nM) (first exposure). After 30 mins, cells were washed twice with cold medium and then further incubated at 37°C in the absence or presence of *Cj*-CDT or *Cj*-CdtB (second exposure). 24 h after the initial intoxication, cells were harvested and evaluated for cell cycle arrest progression. The data were combined from 3 independent biological replicates (n=3) and represent the percentage of cells within the monolayer arrested at the G_2_/M interface. Error bars represent standard deviations. **(A)** The data were fit to a log [Cj-CDT] vs response equation on GraphPad Prism (version 8.1.2). The CCA_50_ (i.e., EC_50_) value was determined for *Cj*-CDT (ABC) but not the binary subunit combinations. R^2^ values indicate fit of the data to the regression model. **(B, C)** Statistical analyses of the data were conducted using one-way Anova, followed by Tukey’s multiple comparisons test. P < 0.05 indicates statistical significance (α = 0.05), “ns” indicates differences were not statistically significant. **(D–F)** Equimolar concentrations of *Cj*-CdtA, *Cj*-CdtB, and *Cj*-CdtC (all 10 μM), were incubated together on **(D)** ice or **(E)** at 37°C. After 1 h, the mixture was diluted in PBS pH 7.4 (to final concentrations of 10 μM, 5 μM, 1 μM, 100 nM, and 10 nM), and then dialyzed at 37°C using a 50 kDa molecular weight cutoff dialysis membrane at a 1:1000 sample-to-dialysis buffer volume ratio. After 48 h, the dialyzed toxin was evaluated for holotoxin complex retention using immunoblot analysis, by employing an antibody specific for *Cj*-CdtB. The ratios of the input mixtures and corresponding recovered retentates of the same concentration were independently compared for each concentration using densitometric analyses, indicated above the blot. (*i.e.*, for the 10 μM *Cj*-CdtB concentration we compared non-dialyzed input to dialysis retentate). For the 5 μM *Cj*-CdtA/B/C sample, the variance (between the 3 independent biological replicates) of the ratio between non-dialyzed sample to dialysis retentate was calculated as **(D)** ± 0.06 and **(E)** ± 0.09. For the 10 μM *Cj*-CdtA/B/C sample, the variance of the ratio between non-dialyzed sample to dialysis retentate was calculated as **(D)** ± 0.05 and **(E)** ± 0.14. **(F)** Dialysis of *Cj*-CdtA, *Cj*-CdtB, and *Cj*-CdtC subunits that were refolded concurrently together (denatured subunits refolded concurrently) was compared to mixed *Cj*-CDT subunits (denatured subunits refolded individually). Data shown are representative of 3 independent biological replicates (n =3).

The active form of *Cj*-CDT is generally considered to constitute a tripartite complex comprising equimolar *Cj*-CdtA, *Cj*-CdtB, and *Cj*-CdtC (Lee et al., 2003; Gargi et al., 2012), which is a model that has emerged primarily from high resolution structural data obtained for *Aa*-CDT and *Hd*-CDT (Dragana Nesic and Stebbins, 2004; Yamada et al., 2006) and previous functional studies of *Cj*-CDT (Lara-Tejero and Galan, 2001). To experimentally evaluate this widely accepted model, we conducted experiments to compare the concentrations at which mixtures of *Cj*-CdtA, *Cj*-CdtB, and *Cj*-CdtC are assembled into a tripartite structure and induce arrest of cell cycle progression at the G_2_/M interface within human colonic intestinal epithelial-derived HCT116 cells. Congruent with previous reports (Lara-Tejero and Galan, 2001; Lee et al., 2003), our studies revealed that *Cj*-CDT-dependent arrest of cell cycle progression occurs in a dose-dependent manner (**Figure 1A**). Moreover, maximal cellular activity requires all three CDT subunits. The concentration of *Cj*-CDT subunit mixtures required to arrest approximately 50% of HCT116 cells within a monolayer (*i.e.*, CCA_50_) was experimentally determined to be approximately 1 nM (**Figure 1A**). Notably, this concentration was >10,000-fold lower than the toxin concentration used to generate crystals suitable for collecting high-resolution structural data for *Aa*-CDT and *Hd*-CDT. These results were comparable to those obtained with preassembled recombinant toxin (CCA_50 _= 2.8 (± 1.0) nM), which is prepared by concurrently refolding together equimolar concentrations of purified and denatured recombinant *Cj*-CdtA, *Cj*-CdtB, and *Cj-*CdtC subunits. Arrest of Cell cycle progression was observed in cells exposed to mixtures of *Cj*-CdtA, *Cj*-CdtB, and *Cj*-CdtC (each at 10 nM) for only 60 min, which was comparable to cells that had been continuously exposed to the toxin for 24 h (**Figure 1B**). These results are consistent with previous work (Whitehouse et al., 1998) showing that within the first hour of exposure to *Cj*-CDT, the toxin had sufficiently bound and been internalized into host cells, resulting in DNA damage, induction of the DNA damage response, and arrest of cell cycle progression.

Additional studies to assess whether concurrent exposure of HCT116 cells to mixtures of *Cj*-CdtA, *Cj*-CdtB, and *Cj*-CdtC is essential for toxin cellular activity, revealed that prebinding of *Cj*-CdtA and *Cj*-CdtC, followed by removal of unbound subunits, and subsequent addition of *Cj*-CdtB, resulted in detectable arrest of cell cycle progression, albeit to a lesser degree than cells exposed to equimolar mixtures of the three subunits (**Figure 1C**). While these studies did not reveal the mechanism of cellular intoxication in the absence of concurrent Cj-CDT subunit exposure, the results are consistent with a conclusion that concurrent administration of *Cj*-CdtA, *Cj*-CdtB, and *Cj*-CdtC to cells is not essential for intoxication.

### High molecular weight *Cj*-CDT tripartite structures are not captured during dialysis at low concentrations of toxin sufficient to induce arrest of cell cycle progression

To evaluate the importance of the predicted tripartite *Cj*-CDT structure for toxin activity, we next determined the concentrations at which mixtures of *Cj-*CdtA, *Cj*-CdtB, and *Cj*-CdtC are retained within dialysis tubing. Equimolar concentrations of *Cj-*CdtA, *Cj*-CdtB, and *Cj*-CdtC (between 0.01 and 10 μM) were premixed on ice. After 1 h, the subunit mixtures were transferred to dialysis membrane cassettes (50 kDa molecular weight cutoff (MWCO)), which were then incubated in dialysis buffer at a 1:1000 volume ratio of sample to dialysis buffer. After 48 h, the retentates were collected, and analyzed versus the corresponding input (*i.e.*, non-dialyzed) mixtures of *Cj-*CdtA, *Cj*-CdtB, and *Cj*-CdtC. Based on the molecular mass cutoff of 50 kDa, we predicted that *Cj-*CdtA (29.9 kDa), *Cj*-CdtB (29.4 kDa), and *Cj*-CdtC (21.4 kDa), if assembled into a heterotrimeric complex (with a calculated molecular mass of approximately 80.7 kDa), would be recoverable from the dialysis membrane. In contrast, we predicted that if mixtures of *Cj*-CdtA, *Cj*-CdtB, and *Cj*-CdtC failed to assemble, then the subunits would diffuse out from the dialysis membrane and not be detected within the dialysis retentate. For these studies, we used immunoblot analyses to compare the relative levels of *Cj*-CdtB in both the input mixtures and corresponding recovered retentates of the same concentration, under the premise that *Cj*-CdtB would be recovered within the retentate when in complex with *Cj-*CdtA and *Cj*-CdtC, but not if the subunit was present as a non-associated monomer. To compare the relative recovery of *Cj*-CdtB more easily within individual dialysis retentates, each of the input mixtures and recovered retentate samples were equally diluted to a final theoretical concentration of 0.01 μM, as a point of comparison against the non-dialyzed input *Cj*-CdtB sample at 0.01 μM (**Figure 1D**). As an example, for the 1 μM condition, both the non-dialyzed and dialyzed samples were diluted 100-fold prior to immunoblot analysis. These experiments revealed that *Cj*-CdtB was not detected within the retentates of dialyzed subunit mixtures at concentrations of 0.01, 0.1, or 1.0 μM (**Figure 1D**). However, *Cj*-CdtB was detected within the retentates of *Cj*-CDT subunit mixtures dialyzed at concentrations of 5 or 10 μM (**Figure 1D**). The same degree of *Cj*-CdtB retention within the dialysis tubing was observed in studies where the preincubation of equimolar mixtures of *Cj*-CDT subunits was conducted at 37°C (**Figure 1E**). Similar results were also obtained using preassembled toxin, which is toxin prepared by concurrently refolding purified and denatured recombinant forms of *Cj*-CdtA, *Cj*-CdtB, and *Cj*-CdtC subunits (**Figure 1F**). These data suggest that, at the lowest concentrations of toxin that are sufficient to induce arrest of cell cycle progression, *Cj-*CdtA, *Cj*-CdtB, and *Cj*-CdtC interact with cell monolayers predominantly as mixtures of non-interacting subunit monomers.

### MST reveals low affinity interactions between *Cj*-CDT subunits

**Figure 2:**
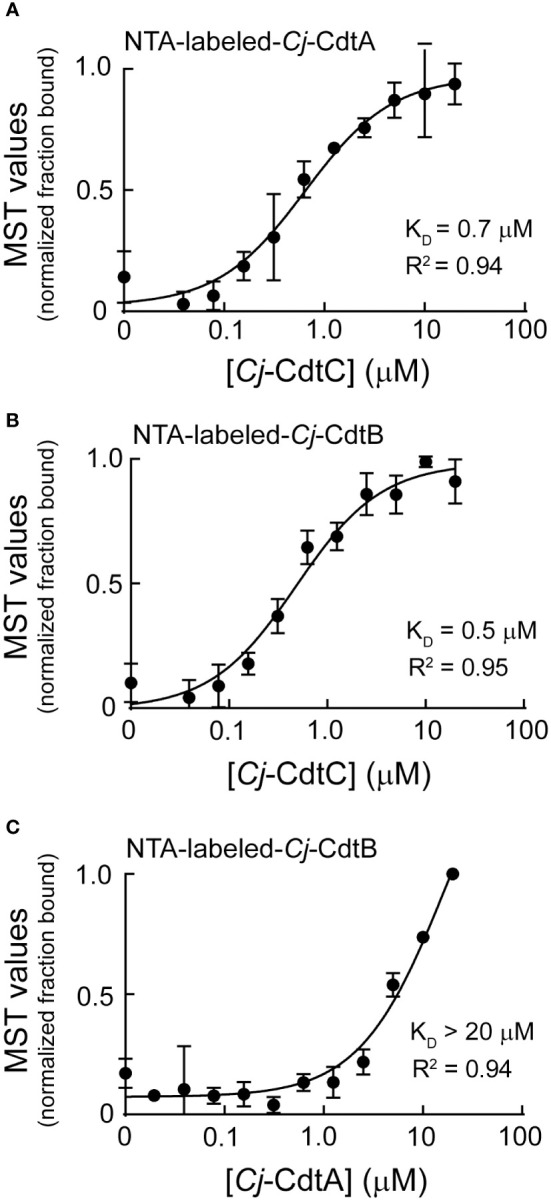
Microscale thermophoresis analysis of *Cj*-CdtA, *Cj*-CdtB, and *Cj*-CdtC subunit interactions. NTA fluorescent labeled *Cj*-CdtA or *Cj*-CdtB subunits were incubated at 37°C with the indicated unlabeled *Cj*-CDT subunit(s) (with polyhistidine tags removed) as follows: **(A)** NTA-labeled-*Cj*-CdtA (0.1 μM) + unlabeled-*Cj*-CdtC (0.04-20 μM), **(B)** NTA-labeled-*Cj*-CdtB (0.1 μM) + unlabeled-*Cj*-CdtC (0.04-20 μM), and **(C)** NTA-labeled-*Cj*-CdtB (0.1 μM) + unlabeled-*Cj*-CdtA (0.02-20 μM), as indicated on the graphs. After 1 h, subunits were evaluated for binding at 37°C using microscale thermophoresis. The data on the graph were combined from 3 independent biological replicates (n =3). The data were fit to a log [*Cj*-CDT] vs response equation on GraphPad Prism 8.1.2. The K_D_ was derived from each binding curve. R^2^ values indicate fit of the data to the regression model.

Dialysis retention experiments described immediately above (**Figure 1D**) suggested that at low nanomolar concentrations, biologically active *Cj*-CDT exists primarily as mixtures of non-associated monomers of *Cj-*CdtA, *Cj*-CdtB, and *Cj*-CdtC, complicit with the idea that *Cj*-CDT subunits interact with relatively low affinities. To evaluate Cj-CDT subunit interactions more quantitatively, we employed microscale thermophoresis (MST) (Wienken et al., 2010). Overall, these studies indicated that *Cj*-CDT subunit interactions (*i.e.*, *Cj*-CdtA and *Cj*-CdtB, *Cj*-CdtA and *Cj*-CdtC, *Cj*-CdtB and *Cj*-CdtC) occur with relatively low affinity (**Table 1**) and were not detectable by MST at concentrations at which the toxin induces arrest of cell cycle progression (*i.e.*, 1-10 nM) (**Figure 1A**). Sigmoidal, saturable binding curves were obtained for mixtures of *Cj*-CdtA and *Cj*-CdtC as well as mixtures of *Cj*-CdtB and *Cj*-CdtC, with dissociation constants (K_D_) of approximately 0.7 μM and 0.5 μM, respectively (**Figures 2A, B**). *Cj*-CdtA interactions with *Cj*-CdtB occurred with even lower affinity, as MST measurements yielded an apparent K_D_ of >20 μM (**Figure 2C**). Non-sigmoidal, non-saturable binding curves were obtained from experiments using NTA-565 dye-labeled *Cj*-CdtC, suggesting that labeling of *Cj*-CdtC interfered with interactions between *Cj*-CdtA and *Cj*-CdtB. Our MST data suggest aberrant binding between amino-terminal labeled *Cj*-CdtC and *Cj*-CdtA. The source of these aberrant binding data cannot be readily explained, as high-resolution structural data are not yet available for *Cj-*CDT. However crystal structures of the tripartite structures of *Hd*-CDT and *Aa*-CDT, reveal that the amino-terminus of the CdtC subunit contacts both the CdtA and CdtB subunits at the interdomain surfaces present in the assembled tripartite structures of both these toxins. From these structures, it’s reasonable to predict that alterations in the interdomain spanning CdtC amino-terminal peptide might impact the stability of the assembled heterotrimer. Nonetheless, it’s not clear whether the relevance of these interdomain interactions of the CdtC subunit carboxyl termini of *Hd*-CDT and *Aa*-CDT extends to *Cj*-CdtC as well. Overall, these data are consistent with those obtained using dialysis retention, where at the lowest concentrations of toxin found to be sufficient to induce arrest of cell cycle progression, mixtures of *Cj-*CdtA, *Cj*-CdtB, and *Cj*-CdtC consist primarily of non-interacting, subunit monomers.

**Table 1:** *Cj*-CDT Microscale thermophoresis table. .

MST-derived *Cj*-CDT subunit binding parameters^a^				
subunit combinations^b^	binding affinity^c^	first concentration of titrant yielding detectable signal above background^d^	saturable binding^e^	R^2f^
A + B* A* + C B* + C	>20 μM 0.65 (+/- 0.1) μM 0.48 (+/- 0.1) μM	2.0 μM 0.31 μM 0.15 μM	no yes yes	0.95 0.95 0.93

a NTA fluorescent dye labeled Cj-CdtA or Cj-CdtB were incubated with unlabeled *Cj*-CDT subunits (no *, 0.02 – 20 μM) and evaluated for binding using MST.

b A = *Cj*-CdtA, B = *Cj*-CdtB, C = *Cj*-CdtC, * = NTA labeled subunit.

c binding affinity derived from a nonlinear regression model fitted to an equation, describing dose (log (*Cj*-CDT)) vs response (fraction bound).

d concentrations of titrant at which MST signals above background were first observed.

e observation of a clear saturated binding curve for the indicated subunit interaction combination.

f R^2^ values indicate fit of the data to the regression model.

### Immunoprecipitation of *Cj*-CDT tripartite complex

**Figure 3:**
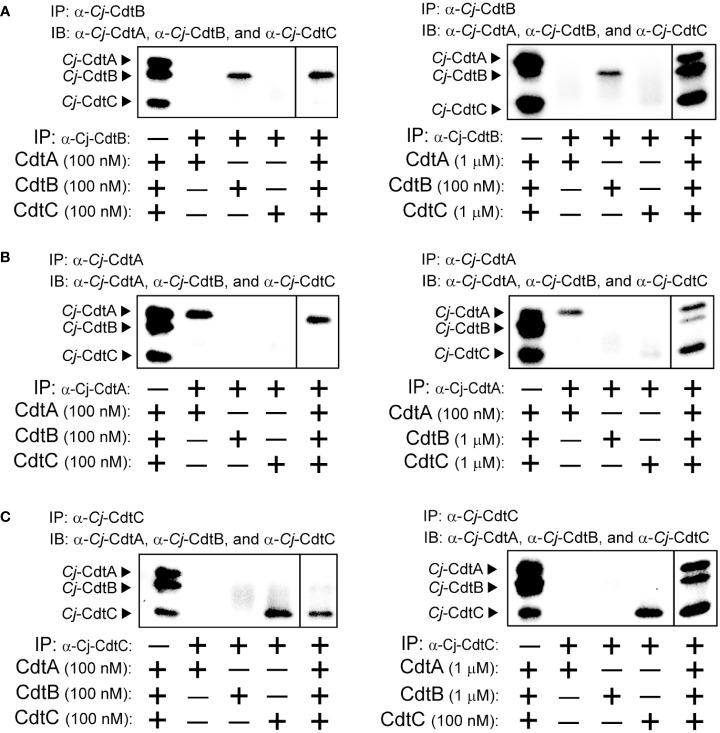
Co-immunoprecipitation of *Cj*-CdtA, *Cj*-CdtB, and *Cj*-CdtC. Immunoprecipitation (IP) was conducted as follows: **(A)** Mixtures of purified *Cj*-CdtA, *Cj*-CdtB, and *Cj*-CdtC (at 100 nM) (panel on the left), or, mixtures of purified *Cj*-CdtB and *Cj*-CdtC (at 1 μM) and *Cj*-CdtA (at 100 nM) were incubated together on ice (panel on the right). After 1h, the mixtures were incubated with α-*Cj*-CdtA antibody bound protein A Dynabeads at 4°C. The next day, the beads were washed, and the bound subunit(s) was/were then eluted and evaluated using immunoblot **(IB)** analysis, probing with antibodies specific for *Cj*-CdtA, *Cj*-CdtB, or *Cj*-CdtC subunit. This process was repeated using (B) purified *Cj*-CdtA, *Cj*-CdtB, and *Cj*-CdtC (at 100 nM) or, mixtures of purified *Cj*-CdtA and *Cj*-CdtC (at 1 μM) and *Cj*-CdtB (at 100 nM), incubated with α-*Cj*-CdtB antibody bound protein A Dynabeads at 4°C. Finally, **(C)** purified *Cj*-CdtA, *Cj*-CdtB, and *Cj*-CdtC (at 100 nM) or, mixtures of purified *Cj*-CdtA and *Cj*-CdtB (at 1 μM) and *Cj*-CdtC (at 100 nM), incubated with α-*Cj*-CdtC antibody bound to protein A Dynabeads at 4°C. Each immunoblot presents data, at identical exposure times, from a single experiment representative of results collected from 3 independent biological replicates (n = 3). The dividing line in each image indicates data that were not directly relevant to the figure, and were therefore spliced out. Data shown are representative of 3 independent biological replicates (n =3).

To more directly assess the capacity of *Cj*-CDT subunits in solution to assemble into tripartite complexes, we examined the recovery of *Cj-*CdtA, *Cj*-CdtB, and *Cj*-CdtC from co-immunoprecipitation experiments using antibodies specifically targeting *Cj-*CdtA, *Cj*-CdtB, or *Cj*-CdtC. In experiments using 0.1 μM mixtures of *Cj*-CdtA, *Cj*-CdtB, and *Cj*-CdtC, immunoprecipitation of *Cj*-CdtB resulted in the recovery of *Cj*-CdtB, but neither *Cj*-CdtA nor *Cj*-CdtC, indicating that detectable complex had not formed (**Figure 3A**). In contrast, immunoprecipitation of *Cj*-CdtB from mixtures containing *Cj*-CdtB (at 0.1 μM) with 10-fold higher concentrations of either *Cj*-CdtA, and *Cj*-CdtC (both at 1.0 μM), revealed the detectable recovery of all three subunits (**Figure 3A**), consistent with the idea that *Cj*-CdtA and *Cj*-CdtC were recovered as part of the heterotrimeric complex with *Cj*-CdtB. Likewise, at the higher 1.0 μM subunit concentrations, *Cj*-CdtB and *Cj*-CdtC were recovered from coimmunoprecipitation experiments using an antibody targeting *Cj*-CdtA (**Figure 3B**). Finally, again at 1.0 μM subunit concentrations, *Cj*-CdtA and *Cj*-CdtB were recovered from coimmunoprecipitation experiments using an antibody targeting *Cj*-CdtC (**Figure 3C**). When taken together with the dialysis retention and MST results described above, the findings from our coimmunoprecipitation studies that heterotrimeric complexes were only recovered when using higher subunit concentrations, further support the idea that, at the lowest concentrations of toxin found to be sufficient to induce arrest of cell cycle progression, mixtures of *Cj-*CdtA, *Cj*-CdtB, and *Cj*-CdtC consist primarily of non-interacting subunit monomers.

## Discussion

The studies described herein were designed to address one of the most poorly understood aspects of CDT biology, which is the relationship between CDT holotoxin structure and toxin cellular activity. Work conducted using CDTs from several mucocutaneous human pathogens have repeatedly demonstrated that all three toxin subunits, CdtA, CdtB, and CdtC, are necessary for maximal toxin cellular activity (Lara-Tejero and Galan, 2001; Shenker et al., 2004; Dixon et al., 2015). High resolution structural data for *Aa*-CDT and *Hd*-CDT indicated that, at high toxin concentrations exceeding 100 μM, the three toxin subunits assemble into a triangle-shaped tripartite structure with each CDT subunit in direct contact with the other two subunits (Dragana Nesic and Stebbins, 2004; Yamada et al., 2006). Structurally inspired mutations, designed to potentially interfere with subunit-subunit interactions, were reported to attenuate cellular activity for *Aa*-CDT (Cao et al., 2005; Yamada et al., 2006). Collectively, these results have contributed to the emergence of a widely-accepted model that CDT binds to and intoxicates sensitive host cells as an assembled tripartite toxin (Gargi et al., 2012). Nonetheless, the biologically active structure of toxin that binds to the surface of sensitive host cells remains poorly understood. Here, we addressed this gap in knowledge by experimentally comparing the concentrations of *Cj*-CDT subunits required for both toxin biological activity, and the assembly of *Cj*-CdtA, *Cj*-CdtB, and *Cj*-CdtC into a heterotrimeric structure. Three independent experimental approaches revealed that solution mixtures of *Cj*-CDT, at the lowest concentrations at which the toxin is biologically active, are comprised primarily of non-interacting subunit monomers. These results bring into question the existing model that *Cj*-CDT cellular intoxication is initiated through interactions of an assembled tripartite toxin, as the biologically active form required for cell surface interactions.

The binding of intracellular-acting AB exotoxins to the plasma membrane of sensitive cells is critical for defining the cell and tissue tropism for specific toxins (Blanke, 2006). As such, a thorough understanding of the molecular determinants by which CDTs recognize and bind to the cell surface is necessary for the development of strategies to mitigate the consequences of toxin action during infection. The experimental observation that both *Cj*-CdtA and *Cj*-CdtC are required for maximal toxin cellular activity (**Figure 1A**), is consistent with the prevailing model that these two subunits together comprise the B component of *Cj*-CDT, which is responsible for the binding of the catalytically active A component, *Cj*-CdtB, to the surface of sensitive host cells. However, studies to identify the *Cj*-CDT subunits that bind to the plasma membrane of sensitive cells revealed that both *Cj*-CdtA and *Cj*-CdtC, but not *Cj*-CdtB, were able to bind independently and in the absence of the other subunits (Lee et al., 2003). Similar observations have also been made for *Aa*-CDT (Boesze-Battaglia et al., 2006), *Ec*-CDT (McSweeney and Dreyfus, 2005), and *Hd*-CDT (Robb Huhn et al., 2021). Taken together, these results suggest the possibility that active toxin complexes need not be preassembled to productively interact with host cells, but instead may assemble directly at the surface of host cells in a sequential manner involving initial binding of CdtA and/or CdtC as a requisite step preceding CdtB binding. In addition, because both CdtA and CdtC are required for maximal cellular activity of all the characterized CDTs, and each subunit can independently bind to the plasma membrane of host cells, we speculate that CdtA and CdtC may contribute in disparate ways to the binding, uptake, and intracellular trafficking of CdtB. This, in fact, has been reported for *Aa*-CDT, *Ec*-CDT and *Hd*-CDT (Damek-Poprawa et al., 2012; Dixon et al., 2015).

The exclusive use of recombinant forms of *Cj*-CDT subunits in the work described here and from other groups (Lara-Tejero and Galan, 2001; Lee et al., 2003) stems from the notoriously low levels of the toxin recovered from culture supernatants of *C. jejuni*. The successful purification or concentration of secreted *Cj*-CDT from *C. jejuni* has not been reported. In our laboratory, toxin cellular activity within liquid or biphasic cultures (in which bacteria are cultivated within a thin layer of liquid medium overlaying the surface of solid agar plates) is detectable at levels equivalent to recombinant toxin at low or sub-nanomolar levels. Detection of individual subunits by immunoblot analysis is difficult, suggesting that active toxin is present in culture filtrates at subnanomolar levels.

The structure of functional *Cj*-CDT generated and released by the bacterium prior to intoxication of host cells has not been definitively resolved. One model is that the tripartite complex of the toxin is assembled prior to release into the extracellular environment, possibly following translocation of *Cj*-CdtA, *Cj*-CdtB, and *Cj*-CdtC across the inner membrane from the cytosol to the periplasmic space, which promotes protein folding and assembly of multi-component proteins (Miller and Salama, 2018). Indeed, cholera and pertussis toxins, both multi-subunit toxins, are believed to be secreted following assembly in the periplasm of the pathogens that generate these toxins (Sandkvist et al., 2000; Burns, 2021). In contrast, the multi-component anthrax toxins (Collier and Young, 2003) and Iota toxin from *Clostridium perfringens* (Sakurai et al., 2009) are secreted as individual subunits, which assemble only after secretion. In the case of the anthrax lethal and edema toxins, the individual components assemble on the surface of host cells. An important gap in knowledge in *Cj*-CDT biology remains the mechanisms by which the individual CDT subunits are folded, assembled, and released into the extracellular environment. Interestingly, genes homologous to those typically found in type II secretion systems, which facilitate secretion of a variety of toxins to the extracellular environment, have not been identified in the genomes of *C. jejuni*, as well as other ε-proteobacteria (Gabbert et al., 2023). *Cj*-CDT has been reported to be associated with outer membrane vesicles (OMVs) (Lindmark et al., 2009), which are generated and released by many Gram-negative bacteria (Rokas Juodeikis, 2022), although the role of OMVs in the *Cj*-CDT intoxication mechanism remains to be delineated. Nonetheless, the robust biological activity of highly purified recombinant CDT subunits indicates that association with OMVs is not essential for the capacity of the toxin to bind and enter host cell in order to exert genotoxic activity.

In summary, the results of the studies described here suggest that the existing paradigm that *Cj*-CdtA, *Cj*-CdtB, and *Cj*-CdtC functionally interact with host cells as a preassembled, heterotrimeric complex should be revisited. Although the mechanism of how mixtures of *Cj-*CDT subunits interact with sensitive host cells has not been definitively established, our data prompt consideration of alternative models. In particular we speculate that individual subunits assemble into biologically active toxin at the cell surface, probably by a mechanism facilitated by interactions with one or more cell surface receptors. However, because equilibrium binding between subunits occur at very high and perhaps non-physiological concentrations of *Cj*-CdtA, *Cj*-CdtB, and *Cj*-CdtC, we cannot rule out the possibility that the toxin does interact with cells as an assembled heterotrimeric complex, albeit at very low concentrations that are undetectable by experimental approaches used in this study. Notably, such a scenario implies that assembled *Cj-*CDT possess a much higher specific activity than has been previously experimentally determined. Additional work will be required to fully understand how *Cj*-CDT subunits collaborate to carry out cellular intoxication.

## Data availability statement

The raw data supporting the conclusions of this article will be made available by the authors, without undue reservation.

## Ethics statement

Ethical approval was not required for the studies on humans in accordance with the local legislation and institutional requirements because only commercially available established cell lines were used. Ethical approval was not required for the studies on animals in accordance with the local legislation and institutional requirements because only commercially available established cell lines were used.

## Author contributions

HC: Conceptualization, Formal Analysis, Investigation, Methodology, Writing – original draft, Writing – review & editing. CA: Conceptualization, Investigation, Methodology, Writing – review & editing. MC: Conceptualization, Investigation, Writing – review & editing. WB: Conceptualization, Resources, Writing – review & editing. SB: Conceptualization, Funding acquisition, Project administration, Resources, Supervision, Writing – review & editing.
