# Corrigendum: Revisiting bacterial cytolethal distending toxin structure and function

**DOI:** 10.3389/fcimb.2024.1366193

**Published:** 2024-01-16

**Authors:** Henry Chen, Claire J. Ang, Molly K. Crowder, William M. Brieher, Steven R. Blanke

**Affiliations:** ^1^ Department of Microbiology, School of Molecular and Cellular Biology, University of Illinois at Urbana-Champaign, Urbana, IL, United States; ^2^ Department of Cellular and Developmental Biology, University of Illinois at Urbana-Champaign, Urbana, IL, United States; ^3^ Department of Pathobiology, College of Veterinary Medicine, University of Illinois at Urbana-Champaign, Urbana, IL, United States; ^4^ Biomedical and Translational Sciences Department, Carle-Illinois College of Medicine, University of Illinois, Urbana, IL, United States

**Keywords:** AB toxin, cytolethal distending toxin, protein-protein interactions, *Campylobacter jejuni*, DNA damage, holotoxin structure

**Incorrect author name**

In the published article, an author name was incorrectly written as “**Molly Crowder ^1^**”. The correct spelling is “**Molly K. Crowder ^1^**”.

The authors apologize for these errors and state that this does not change the scientific conclusions of the article in any way. The original article has been updated.

**Missing acknowledgements**

In the published article, the acknowledgement section was left out. The correct acknowledgement statement appears below.

**Acknowledgments**

We would like to thank Ms. Ria, Ravi for her early experimental work contributing to the paper that was not included in the final version of the paper.

The authors apologize for these errors and state that this does not change the scientific conclusions of the article in any way. The original article has been updated.

